# One-Pot Synthesis of Sulfur-Doped TiO_2_/Reduced Graphene Oxide Composite (S-TiO_2_/rGO) with Improved Photocatalytic Activity for the Removal of Diclofenac from Water

**DOI:** 10.3390/ma13071621

**Published:** 2020-04-01

**Authors:** Marin Kovačić, Klara Perović, Josipa Papac, Antonija Tomić, Lev Matoh, Boštjan Žener, Tomislav Brodar, Ivana Capan, Angelja K. Surca, Hrvoje Kušić, Urška Lavrenčič Štangar, Ana Lončarić Božić

**Affiliations:** 1 Faculty of Chemical Engineering and technology, University of Zagreb, Marulićev trg 19, HR-10000 Zagreb, Croatia; kperovic@fkit.hr (K.P.); jpapac@fkit.hr (J.P.); atomic@fkit.hr (A.T.); abozic@fkit.hr (A.L.B.); 2 Faculty of Chemistry and Chemical Technology, University of Ljubljana, Večna pot 113, SI-1000 Ljubljana, Slovenia; lev.matoh@fkkt.uni-lj.si (L.M.); bostjan.zener@fkkt.uni-lj.si (B.Ž.); urska.lavrencic.stangar@fkkt.uni-lj.si (U.L.Š.); 3 Division of Material Physics, Ruđer Bošković Institute, Bijenička cesta 54, HR-10000 Zagreb, Croatia; tomislav.brodar@irb.hr (T.B.); ivana.capan@irb.hr (I.C.); 4 Department of Chemistry, National Institute of Chemistry, Hajdrihova 19, SI-1001 Ljubljana, Slovenia; angelja.k.surca@ki.si

**Keywords:** solar photocatalysis, sulfur-doped TiO_2_, TiO_2_-reduced graphene oxide composite, diclofenac

## Abstract

Sulfur-doped TiO_2_ (S-TiO_2_) composites with reduced graphene oxide (rGO), wt. % of rGO equal to 0.5%, 2.75%, and 5.0%, were prepared by a one-pot solvothermal procedure. The aim was to improve photocatalytic performance in comparison to TiO_2_ under simulated solar irradiation for the treatment of diclofenac (DCF) in aqueous medium. The obtained composites were characterized for physical-chemical properties using thermogravimetric analysis (TGA), X-ray diffractograms (XRD), Raman, scanning electron microscopy (SEM)/energy dispersive X-ray (EDX), Brauner Emmett Teller (BET), and photoluminescence (PL) analyses, indicating successful sulfur doping and inclusion of rGO. Sulfur doping and rGO have successfully led to a decrease in photogenerated charge recombination. However, both antagonistic and synergistic effects toward DCF treatment were observed, with the latter being brought forward by higher wt.% rGO. The composite with 5.0 wt.% rGO has shown the highest DCF conversion at pH 4 compared to that obtained by pristine TiO_2_, despite lower DCF adsorption during the initial dark period. The expected positive effects of both sulfur doping and rGO on charge recombination were found to be limited because of the subpar interphase contact with the composite and incomplete reduction of the GO precursor. Consequent unfavorable interactions between rGO and DCF negatively influenced the activity of the studied S-TiO_2_/rGO photocatalyst under simulated solar irradiation.

## 1. Introduction

The prospects of TiO_2_ photocatalysis are immense, ranging from clean energy production to water purification using solar irradiation [1,2]. However, the activity of pristine TiO_2_ under solar irradiation of is hindered by low absorption within the visible spectrum, because of its prohibitively wide bandgap. Second, the performance of TiO_2_ in those cases is likewise hindered by photogenerated charge recombination. Some studies have shown that up to 90% of the photogenerated charges recombine, releasing heat as a byproduct, and hence do not perform useful photocatalytic work [3]. Hence, suppression of charge recombination would further yield an improvement in photocatalytic activity for the processes of interest. Improvements with respect to higher photocatalytic activity under visible irradiation can be achieved by: (i) Deposition of noble metals on the surface of TiO_2_, (ii) doping the crystalline lattice of TiO_2_, (iii) creating composites with other semiconducting materials, and (iv) with dye-sensitizers, providing transfer of the photogenerated electron from or to TiO_2_ [4,5,6,7]. An attractive solution is the implementation of graphene or reduced graphene oxide (rGO) as an electron sink, akin to the function of deposited noble metals, but at a significantly lower cost. High conductivity of graphene and reduced graphene oxide (rGO) facilitates electron transfer, providing decreased recombination rates [8]. Furthermore, rGO can promote photocatalytic activity toward degradation by adsorbing pollutants, thereby increasing the odds of the reaction occurring between the adsorbed pollutant and surface-generated radical species [9]. Graphene oxide (GO) can be reduced using harmful reducing agents or benign agents, such as green tea extract [10,11]. TiO_2_/rGO composites can be prepared by sol-gel and solvothermal procedures with simultaneous TiO_2_ growth and GO reduction to rGO, colloidal blending and ionothermal methods [12,13,14,15]. Sulfur doping can significantly decrease the band gap of TiO_2_ and can promote the inhibition of photogenerated charge recombination, thereby improving photocatalytic activity under solar irradiation. Sulfur doping of TiO_2_ can be achieved by sol-gel, hydrothermal and coprecipitation syntheses [16,17,18,19,20]. In this paper, a sulfur-doped TiO_2_ (S-TiO_2_) and rGO composite with varying wt. % of rGO was obtained in a one-pot solvothermal synthesis. The simultaneous reduction and doping were achieved by in situ generated sulfide ions (S^2−^) from thioacetamide hydrolysis under solvothermal conditions. The photocatalytic performance of immobilized S-TiO_2_/rGO thin films for DCF abatement in water under simulated solar irradiation was investigated.

## 2. Materials and Methods

### 2.1. Preparation of Graphene Oxide (GO)

All chemicals used in this work were of analytical or higher grade. Graphene oxide (GO) was prepared according to the Hummers procedure with minor modifications [21]. Graphite flakes (C, Sigma-Aldrich) were ground and sieved. Total of 3 g of mesh 70 graphite was treated with 69 mL of sulfuric acid (H_2_SO_4_, Honeywell) and 1.5 g of dry sodium nitrate (NaNO_3_, Kemika) in an ice bath. Total of 9 g of potassium permanganate (KMnO_4_, Kemika) was added to the mixture, taking care not to rise the temperature significantly. After 1 h of stirring, demineralized water was slowly added until the temperature reached near boiling. The mixture was then allowed to cool and was subsequently diluted with 1 L of deionized water. Hydrogen peroxide (H_2_O_2_, 30%, T.T.T.) was added to the diluted solution, until the cessation of the observed effervescence. The mixture was centrifuged, and the whitish precipitate was discarded, while the brownish floating product was rinsed with 5% hydrochloric acid (HCl, Kemika). The residual acid was washed until the suspension became nearly pH neutral. The suspension was dialyzed for 14 d with a Spectrum chemical Spectra/Por membrane in ultrapure water. Water from the obtained suspension was removed in vacuum at 35 °C.

### 2.2. Solvothermal Synthesis of TiO_2_ and S-TiO_2_ Reduced Graphene Oxide Composite (S-TiO_2_/rGO)

GO obtained from the previous procedure was added to 96% ethanol (CH_3_CH_2_OH, EtOH, Gram-mol) in order to obtain a suspension of *γ*(GO) = 1 mg mL^−1^ and was subsequently sonicated for 30 min using a Cole Parmer EW-08848 ultrasonic bath (Cole Parmer, Vernon Hills, IL, USA). An appropriate aliquot of the resulting GO/EtOH suspension needed to achieve the resulting 0.5, 2.75, and 5.0 wt.% of the reduced graphene oxide (rGO) was transferred to a 50 mL polytetrafluoroethylene (PTFE) reactor. Afterwards 200 mg ± 5 mg of thioacetamide (CH_3_SNH_2_, TAA, Acros Organics) was added under constant stirring, along with 1 mL of glacial acetic acid (CH_3_COOH, Fluka). Upon dissolution of thioacetamide, a 1 mL aliquot of tetrabutyl orthotitanate (Ti(OCH_2_CH_2_CH_2_CH_3_)_4_, TBT, Acros Organics) was added under stirring. In order to obtain pristine TiO_2_, GO and thioacetamide were omitted from the synthesis. The PTFE reactor was transferred to a stainless steel autoclave and heated to 180 °C for 12 h in a UN-55 laboratory oven (Memmert, Schwabach, Germany). After cooling to room temperature, the product was centrifuged and rinsed successively three times with distilled water. The product slurry was then dried at 60 °C under vacuum.

### 2.3. Immobilization of S-TiO_2_/rGO Composites

The obtained composite was immobilized on round glass substrates (*r* = 37.5 mm) using a low-temperature procedure developed by Kete et al. [22]. The procedure calls for the preparation of a titania precursor sol and silica precursor sol. The titania sol was obtained by perchloric acid (HClO_4_, Kemika) catalyzed hydrolysis of an ethanolic solution of titanium isopropoxide (Ti{OCH(CH_3_)_2_}_4_, Acros Organics) under reflux for 48 h. The silica sol was prepared by hydrochloric acid-catalyzed hydrolysis of tetraethyl orthosilicate (Si(OC_2_H_5_)_4_) in water. Once a clear silica sol was obtained, it was mixed with the titania sol, ethanol, and colloidal silica Levasil 200/30 (Obermeier) to form the binder sol. To the binder sol an appropriate amount of S-TiO_2_/rGO was added and homogenized in an ultrasonic bath for 15 min. The photocatalyst was deposited on glass substrates by spin coating at 1500 rpm for 30 s using a KW-4A spin coater (Chemat Technology). The plates were finally thermally treated at 200 °C in air for 2 h, using an UN-55 programmable laboratory oven (Memmert, Schwabach, Germany).

### 2.4. Characterization of the S-TiO_2_/rGO Composite

Thermogravimetric analysis (TGA) was performed by heating 5 mg of the sample in a Pt pan, within a temperature range of 25 °C to 600 °C at a heating rate of 10 °C min^−1^ in synthetic air. The analysis was performed on a Q500 TGA analyzer (TA Instruments, New Castle, PA, USA).

Morphological features were determined by scanning electron microscopy (SEM) and energy dispersive X-ray (EDX) spectroscopy, using an Ultra Plus SEM (Zeiss, Jena, Germany) equipped with an Oxford X-Max silicon drift detector. The samples were loaded on a graphite adhesive tape, without vapor phase deposition pretreatment.

Brauner Emmett Teller (BET) specific surface area was determined by N_2_ adsorption/desorption isotherms on a Gemini 2380 instrument (Micrometrics, Norcross, GA, USA). The samples were degassed beforehand at 250 °C in vacuum.

X-ray diffractograms (XRD) of the samples were measured with a X’Pert PRO MPD instrument (PANalytical, Almelo, Netherlands), using Cu Kα1 radiation at 2*θ* from 10° to 70° in 0.033° increments. An ICDD PDF standard card no. 21-1272 was used as anatase reference. D-spacing was calculated according to Equation (1):

(1) $$\frac{1}{d^{2}}=\frac{h^{2}+k^{2}}{a^{2}}+\frac{l^{2}}{c^{2}}$$

where *d* represents the interplanar distance, i.e. D-spacing, *h*, *k* and *l* are Miller indices. The size of crystallites was calculated according to the Scherrer Equation (2) [23,24]:

(2) $$\tau =\frac{K\lambda }{\beta \cos\theta }$$

where *τ* represents the calculated crystallite size, *K* is a dimensionless factor related to the shape of crystallites (*K* = 0.9), *β* is the peak width at full maximum (in radians), and *θ* is the Bragg angle. 

Raman spectroscopy was performed on powder samples, using an Alpha300 (Witec, Ulm, Germany) equipped with a microscope and attached atomic force microscope (AFM). A 532 nm laser was used as the excitation source. The integration time was set to 0.5 s, with an average of 100 scans taken.

Photoluminescence (PL) spectra were recorded using a LDM405 405 nm laser diode module (Thorlabs, Newton, NJ, USA) as an excitation source. The emitted PL light was analyzed by a BRC112E CCD array spectrometer (B&W Tek, Newark, NJ, USA).

Diffuse reflectance spectra (DRS) were measured on immobilized thin films by a Lambda 950 UV/Vis spectrometer (Perkin Elmer, Palm Springs, CA, USA), equipped with an integrating sphere. The spectra were recorded at a scan speed of 2063 nm min^−1^, from 2500 nm to 250 nm. The obtained spectra were transformed into the Kubelka–Munk function vs. photon energy in order to obtain band gap values [25].

### 2.5. Investigation of Photocatalytic Activity

Photocatalytic experiments for the degradation of 0.1 mM diclofenac sodium (C_14_H_11_Cl_2_NO_2_Na, DCF, Sigma Aldrich) in water were carried out in a water-cooled batch photoreactor, with the photocatalyst-coated glass plates placed on the bottom of the reactor. The experiments were performed in triplicates. A solar simulator, using a model 69920 power supply, Newport 66921 arc lamp housing fitted with an Osram 450W Xenon arc lamp, and an AM 1.5 G airmass filter (Newport, Irvine, CA, USA) was used to irradiate the photoreactor by a perpendicular, collimated beam of light. A DOS-20 shaker (NeoLab, Heidelberg, Germany) was used to provide mixing within the reactor during the experiments. The pH of the DCF solution was adjusted to 4 ± 0.05, 6 ± 0.05, and 8 ± 0.05 in separate experiments, using dilute HCl and NaOH, before quantitatively transferring 90 mL of the solution into the photoreactor. An initial period of 30 min in the dark was provided to achieve adsorption equilibrium of DCF on the immobilized photocatalyst. After initial pH adjustment and achieving equilibrium in the dark, 1.5 mL of the sample was withdrawn from the reactor and filtered through 0.45 µm syringe filters into vials for high performance liquid chromatographic (HPLC) analysis. Desorption of DCF from the used plates was performed by immersion in an alkaline solution maintained at pH 8 for half an hour, after which the sample was submitted for HPLC analysis. DCF was detected at a wavelength of 276 nm, using isocratic elution with methanol (CH_3_OH, J.T. Baker): 0.1 % formic acid (HCOOH, Fluka) in water = 70:30, on a Nucleosil RP C18 250 mm × 4.6 mm column (Macherey Nagel, Dueren, Germany). The HPLC used was a LC-20 series (Shimadzu, Kyoto, Japan), equipped with a SPD-M20A UV/DAD detector. All stock solutions were prepared from ultrapure water obtained from a Millipore Direct Q3 UV (Merck EMD, Darmstadt, Germany).

## 3. Results and Discussion

### 3.1. Composition and Morphology of the S-TiO_2_/rGO Composite

**Figure 1:**
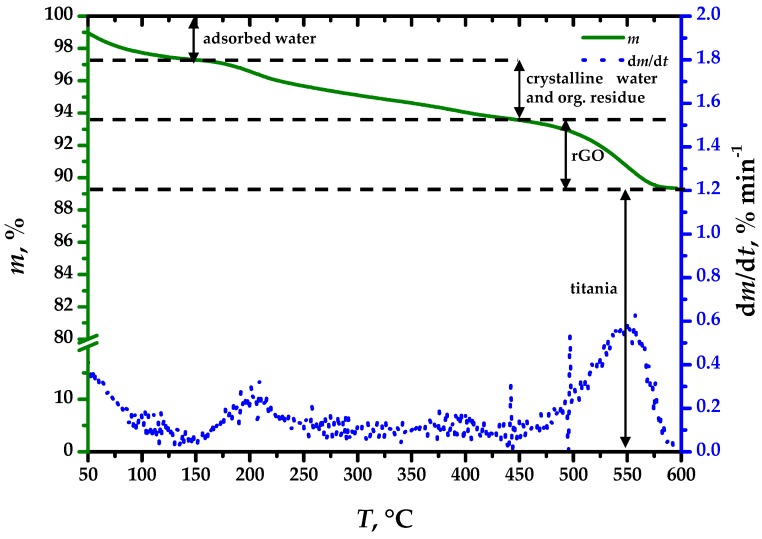
Thermogravimetric analysis of the S-TiO_2_/rGO(5.0%) composite.

The thermogravimetric curve of the 5% wt.(rGO) composite shown in Figure 1 indicates an unremarkable composition. The mass loss up to 150 °C occurred because of evaporation of adsorbed water, amounting to 2.7% of the initial sample mass. Within the 150 °C to 450 °C interval, a loss of crystalline water and organic residue occurs, amounting to 3.8% of the initial mass. Finally, the onset of rGO oxidation at 450 °C amounts to 4.3% of the initial sample mass, indicating successful inclusion of the rGO within the composite.

**Figure 2:**
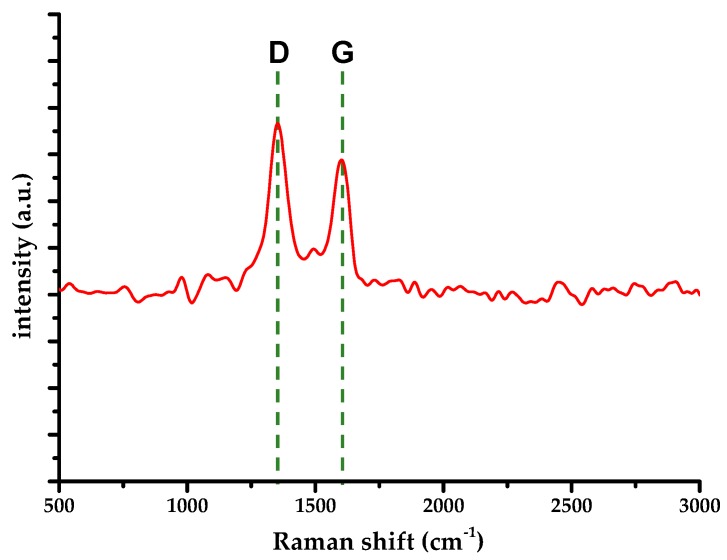
Raman spectrum of S-TiO_2_/rGO(5.0%) composite.

In order to gain further insight of rGO occurrence within the titania matrix, a Raman spectrum analysis of the sample was carried out; results are presented in Figure 2.

The D-mode appears at 1352 cm^−1^, whereas the G-mode occurs at 1600 cm^−1^. The G-mode blueshift indicates the presence of residual functional groups, i.e., incomplete reduction of the starting material [26,27]. Those are presumably carbonyl and carboxyl groups that require C-C bond rupture. Furthermore, a ratio of D-mode and G-mode Raman intensities (*I*_d_/*I*_g_) of about 1.4 was calculated, indicating a relatively large number of defects within rGO, supported by the lack of the 2D-mode and relatively wide G-mode peak. Characteristic Raman modes for anatase have not been detected, i.e., they were not discernable because of the noise floor.

**Figure 3:**
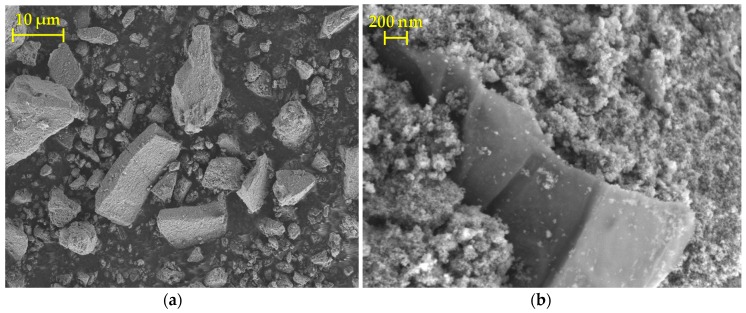
Scanning electron microscopy (SEM) micrographs depicting: (**a**) S-TiO_2_/rGO(5.0%) agglomerates; (**b**) rGO sandwiched in S-TiO_2_ matrix.

The composite appears in the scanning electron micrographs, shown in Figure 3a, as irregular agglomerates, ranging in size from a few micrometers up to several tens of micrometers along the longest dimension. The formation of agglomerates is a consequence of the drying process and subsequent grinding with a pestle and mortar. Upon closer inspection, it can be seen that the S-TiO_2_/rGO(5.0%) sample is composed of nanosized TiO_2_ particles, into which rGO sheets are sandwiched, as shown in Figure 3b. Such morphology can be considered subpar, as phase contact, and hence charge transfer is limited to the near vicinity of the rGO sheets. However, such phase transfer limitations have been noted by other authors applying similar synthesis procedures of the composites [28].

**Figure 4:**
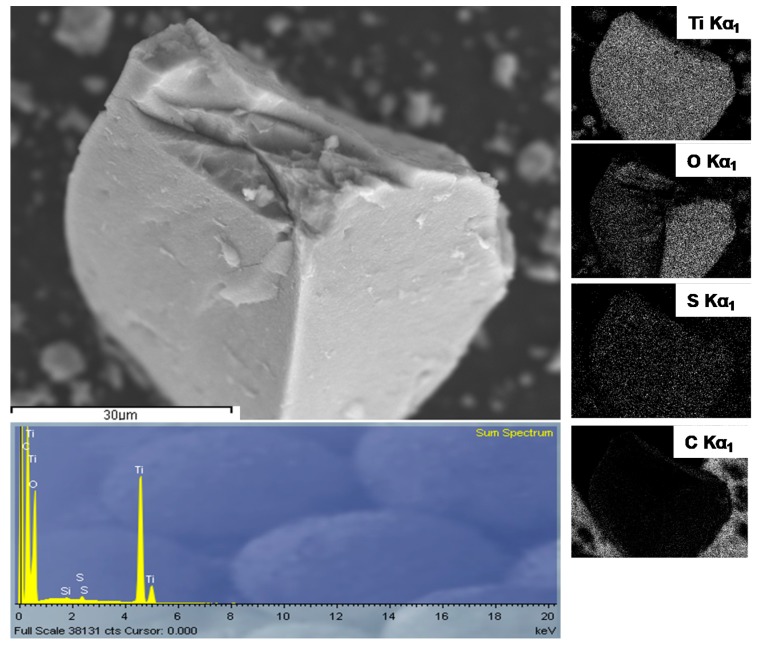
Energy dispersive X-ray (EDX) analysis of S-TiO_2_/rGO(5.0%).

The EDX spectrum, shown in Figure 4, in conjunction with XRD, indicates sulfur doping of TiO_2_ by nano-sulfur formed by reduction of GO with sulfide as a result of TAA hydrolysis.

**Figure 5:**
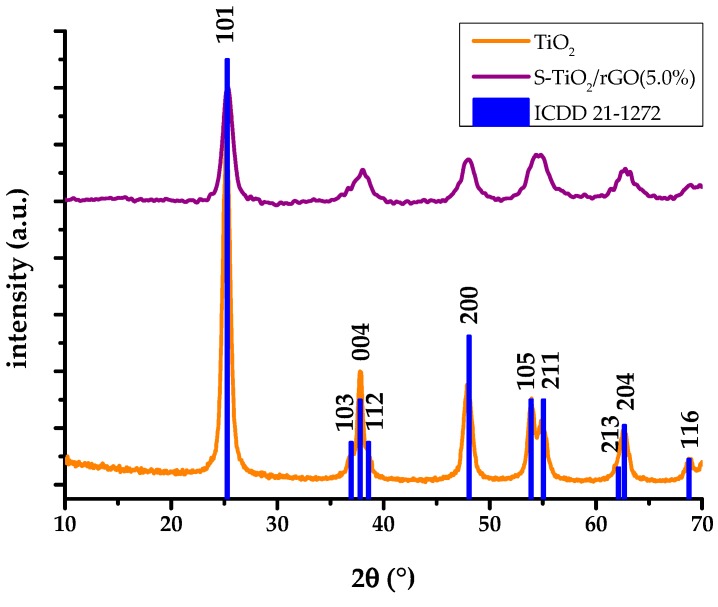
X-ray diffractograms (XRD) analysis of hydrothermally obtained TiO_2_ and S-TiO_2_/rGO(5.0%).

Emission of characteristic X-rays display a uniform distribution of sulfur, along with titanium and oxygen, within the sample. The X-ray diffractogram of the S-TiO_2_/rGO composite with 5% wt.(rGO), presented in Figure 5, shows a qualitatively lesser degree of crystallinity of anatase phase, in comparison to the diffractogram of pristine TiO_2,_ as well as a shift in peak positions, indicating lattice defects caused by the inclusion of sulfur. The qualitative decrease of crystallinity is notable from the deterioration of the peaks in the S-TiO_2_/rGO (5.0%) diffractogram, i.e., the intensity of the 101 *hkl* index peak decreased and the peak has broadened, while the peaks of 103, 112, 105, and 211 *hkl* indices are hardly discernable.

Calculated lattice parameters, cell volume, and crystallite sizes for TiO_2_ and S-TiO_2_/rGO(5.0%) are provided in Table 1.

The results indicate a decrease in cell parameters, and consequently cell volume, because of sulfur doping and the presence of rGO. However, taking into consideration the larger ionic radius of sulfur in comparison to oxygen, such a result is not self-explanatory. Cravanzola et al. [29] studied the effect of sulfur doping on TiO_2_ and concluded that sulfur has exchanged oxygen at the surface of TiO_2_. Gomathi Devi and Kavitha studied the incorporation of S6^+^ into the anatase lattice at Ti4^+^ sites and the subsequent contraction of the cell volume [20]. The overall smaller crystallite domains in S-TiO_2_/rGO (5.0%) are not small enough to cause a blue-shift in the band gap, because of the quantum confinement, which would negatively affect the activity [30]. The effect of the rGO precursor on the crystallinity of anatase is miniscule and could not be detected in the XRD itself, as supported by the literature findings [28,31].

**Figure 6:**
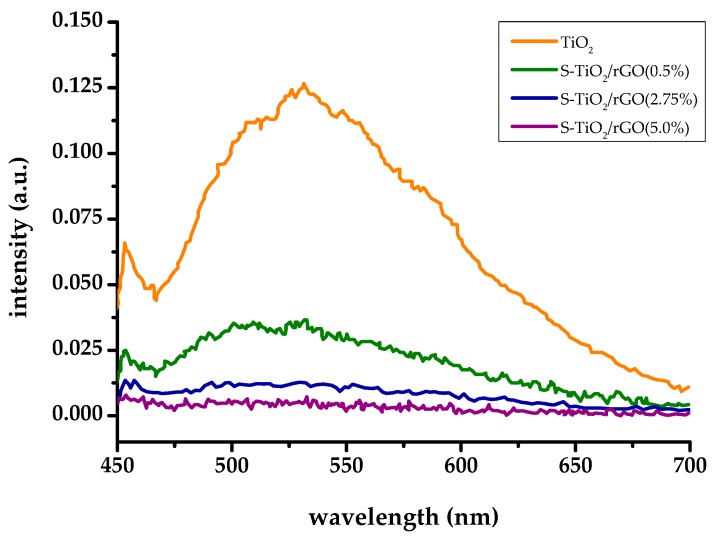
Photoluminescence (PL) spectra of TiO_2_ and S-TiO_2_/rGO composites.

Photoluminescence spectra, shown on Figure 6, indicate a positive, synergistic effect on the inhibition of charge recombination by sulfur doping and the inclusion of rGO within the TiO_2_ matrix, despite relatively poor interphase contact.

The photoluminescence spectrum of TiO_2_ shows a typical peak centered at 531 nm, corresponding to the recombination of the conduction band electron and trapped hole or electron quenching by surface adsorbed oxygen [32]. The inclusion of sulfur doping and rGO has resulted in a remarkable decrease of emission, indicating successful inhibition of recombination. Sulfur doping at shallow impurity levels can trap photo-generated charges, according to Liu et al. [33], which is further contributed by rGO acting as an electron-sink. However, the amount of rGO seems to be the key, as the intensity of measured photoluminescence decreased with the increasing amount of rGO within the S-TiO_2_/rGO composite. However, increasing rGO wt. % from 2.75% to 5% brings diminishing returns. Higher wt. % of rGO leads to an opposite effect, i.e., an increase in recombination, as rGO can behave as a recombination center [8].

**Figure 7:**
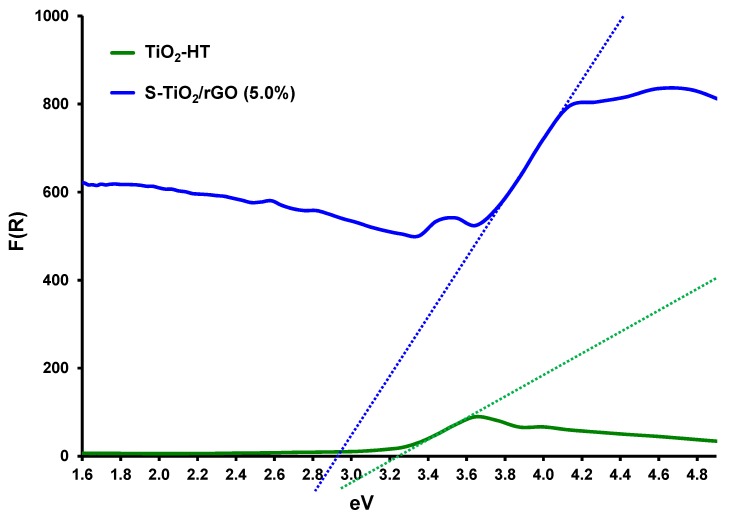
Diffuse reflectance spectra (DRS) spectrum of TiO_2_ [34] and S-TiO_2_/rGO(5.0%).

A further improvement in optoelectronic properties was also noted by DRS, as shown by the Kubelka-Munk transformation of the collected reflectance spectra in Figure 7. 

The band gap (*E*_g_) of the TiO_2_ sample was determined to be 3.21 eV, whereas the band gap for the S-TiO_2_/rGO sample was 2.92 eV. The observed reduction in the band gap is a result of sulfur doping and rGO inclusion. Since the recorded spectra did not differ significantly because of the varying rGO content, they were omitted from Figure 7. Literature findings support the hypothesis that varying rGO content in TiO_2_-based photocatalytic materials does not have a discernible effect on lowering of the bandgap [35,36]. Furthermore, Wang et al. [37] found a similar band gap for sulfur-doped TiO_2_, hence sulfur doping and rGO achieve the same band gap narrowing threshold.

Table 2 shows the BET surface areas of TiO_2_, S-TiO_2_/rGO composites, and rGO. 

The obtained results are comparable to the literature findings, based on similar syntheses and drying procedures for TiO_2_ and rGO respectively [39,40].

The composite with 0.5 wt.% rGO has a lower surface area than pristine TiO_2_, which is to be expected considering the nearly eight-fold difference in specific surface areas of the synthesized TiO_2_ and rGO respectively. However, the composite with 2.75 wt.% rGO has a nearly equal surface area to pristine TiO_2_ and an increase in surface area with 5.0 wt.% rGO in the composite was observed. Such a contribution can be plausibly attributed to the interaction between TiO_2_ and the two-dimensional matt structure of rGO [41].

### 3.2. Photocatalytic Performance of S-TiO_2_/rGO

**Figure 8:**
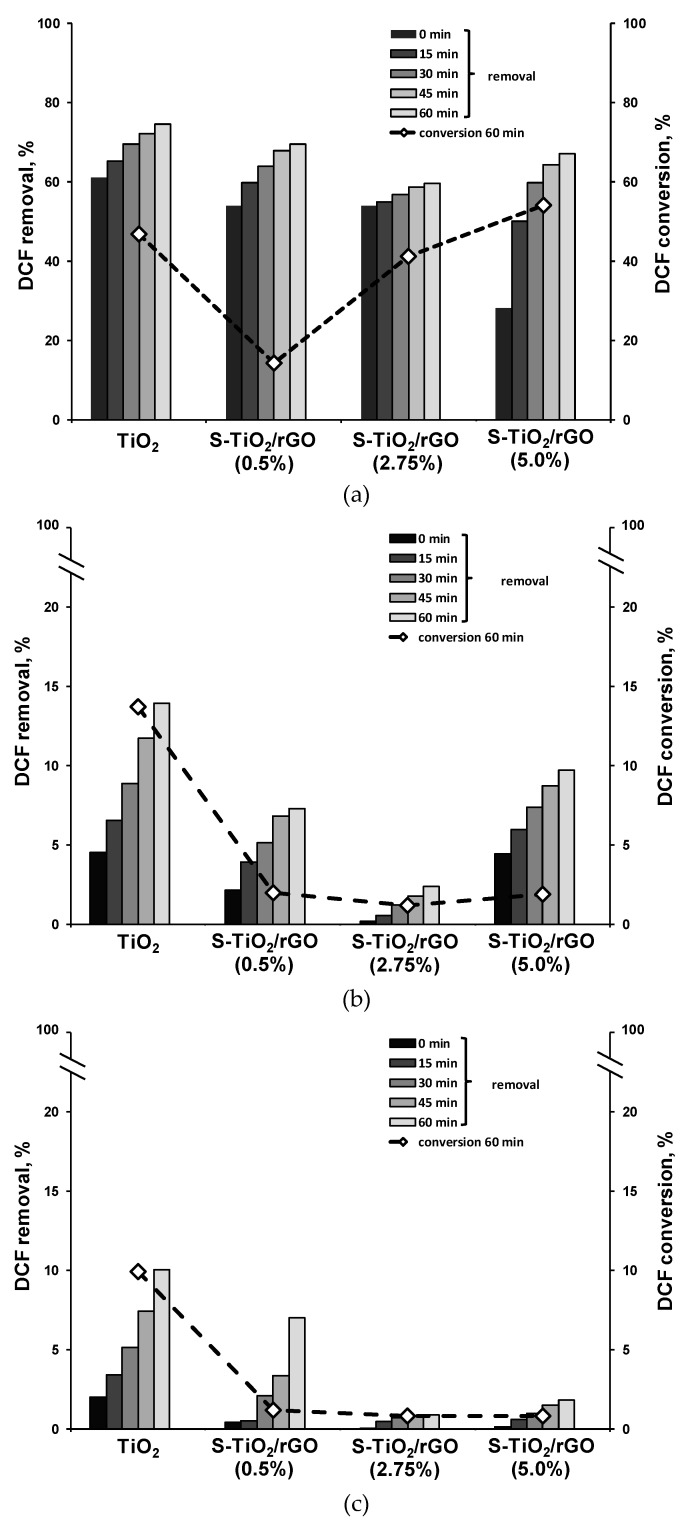
Removal and conversion of diclofenac (DCF) during photocatalytic treatment by solvothermally obtained TiO_2_ and S-TiO_2_/rGO composites at (**a**) pH 4, (**b**) pH 6, and (**c**) pH 8.

Photocatalytic effectiveness of the S-TiO_2_/rGO composites, in comparison to pristine TiO_2_, for the degradation of DCF are compared in Figure 8. Generally, a decrease in removal of DCF, in comparison to TiO_2_, can be noted across all experiments with the S-TiO_2_/rGO composite. The observed effect is pronounced at pH 6 and 8. First, in the case of the 0.5% wt. rGO composite, i.e., S-TiO_2_/rGO(0.5%), the decrease can be attributed primarily to a lower specific surface area, in comparison to pristine TiO_2_, as shown in Table 2. The surface area of the aforementioned composite is 4.1% lower than pristine TiO_2_ and has achieved nearly 5.6% lower DCF removal after treatment. However, in the case of higher wt.% of rGO, the surface area reasoning for the observed effects does not stand true. rGO contains carboxylic groups, resulting in p*K*_a1_ of about 4.0, similar to p*K*_a_ of DCF and p*K*_a2_ of 6.0 [42]. Hence at pH 4 and less acidic conditions, repulsion interactions likely occur between rGO and DCF, as they both contain the same deprotonated carboxyl group moiety. π-π interactions between rGO and DCF, which could possibly offset such an effect, are presumably limited because of a significant number of defects within the obtained rGO. Hence, rGO has not shown a beneficial effect toward DCF adsorption, which would promote photocatalytic degradation. The effectiveness of the rGO within the prepared composite is thereby hindered two-fold, first by subpar morphology, and second, by the defects in the structure of rGO, diminishing the supposed advantages a graphene-like material should offer.

The effects of doping TiO_2_ with sulfur and combining with rGO are seemingly conflicting in terms of photocatalytic activity. Drastically lower conversion was achieved by the 0.5 wt.% composite, i.e., 14.3% vs. 46.8% achieved by pristine TiO_2_ respectively, despite improved inhibition of photo-generated charge recombination in all cases, as previously discussed in terms of PL measurement. Furthermore, the 2.75 wt.% rGO composite has a nearly equal surface area to pristine TiO_2_, but has achieved 15.0% less removal after 60 minutes of treatment, whereas initial removal in the dark was on par to 0.5 wt.% rGO. Most probably sulfur-doping, and inclusion of rGO at lower amounts, significantly altered the band edge positions and hence hindered the direct surface degradation of DCF. Increasing wt.% rGO has a markedly positive effect on the conversion of DCF, because of significantly improved recombination inhibition and increased surface area, hence promoting adsorption onto S-TiO_2_. The beneficial effect on the composite is even more profound, considering that an increasing amount of rGO in fact lowers the amount of the photocatalytically active component, i.e., TiO_2_. At pH 4, the S-TiO_2_/rGO 5 wt.% composite achieved 7.33% higher conversion than TiO_2_, despite lower adsorption due to unfavorable electrostatic interactions. At pH 6 and pH 8, the surface of the composite S-TiO_2_/rGO photocatalyst is negatively charged, resulting in a significant decrease in DCF removal and consequent conversion. It is to be expected, that a shift to a sandwich type composite could yield markedly improved activity, as the phase transfer should be significantly better, therefore improving photogenerated charge separation. Furthermore, the usage of a more pristine graphene-like material should be investigated, in order to assess the impact of incomplete reduction and structural defects.

## 4. Conclusions

The one-pot simultaneous solvothermal reduction of GO to rGO and production of TiO_2_ yielded agglomerates of TiO_2_ with sandwiched rGO sheets. EDX analysis indicated effective doping of TiO_2_ with sulfur, a product of GO reduction by sulfide ions. The obtained morphology can be considered subpar, as the interphase contact between rGO and TiO_2_ does not result in effective photogenerated charge separation. The photocatalytic activity of S-TiO_2_/rGO composite for DCF removal and conversion strongly depends on the wt.% of rGO. The 5 wt. % rGO showed improved photocatalytic activity, i.e., conversion in comparison to the benchmark TiO_2_, despite a lower removal during the initial dark period. The improved activity can be attributed to effective photogenerated charge separation, as measured by a decrease in PL. Whereas the composite with 0.5 wt. % showed significantly worse activity. rGO has shown to have an antagonistic effect on DCF adsorption due to the incomplete reduction, yielding carboxylic and carbonyl groups that repel DCF. The photocatalytic activity is further hindered by the morphology of the composite, which limits the positive effects of charge transfer between TiO_2_ and rGO. At lower wt. % of rGO, the photocatalytic activity is also most likely hindered because of a shift in the band edge position, thus limiting the direct degradation of DCF on the photocatalyst’s surface.

## Figures and Tables

**Table 1:** Comparison of lattice parameters, d-spacings, cell volumes, and crystallite sizes for TiO_2_ and S-TiO_2_/rGO (5.0%).

Sample	*a*, Å	*c*, Å	*d*_101_, Å	*d*_004_, Å	*d*_200_, Å	Cell Volume, Å^3^	*τ*, nm
TiO_2_	3.798	9.515	3.531	2.379	1.899	137.26	17.5
S-TiO_2_/rGO(5.0%)	3.787	9.454	3.516	2.363	1.894	135.56	13.4

**Table 2:** Surface areas of TiO_2_ and S-TiO_2_/rGO composites determined by Brauner Emmett Teller (BET) analysis, with pristine TiO_2_ being denoted as *w*(rGO) = 0% and rGO with *w*(rGO) = 100%.

*w*(rGO), %	BET Surface Area, m^2^ g^−1^
0.0	128.4 ± 1.9 [38]
0.5	123.2 ± 1.6
2.75	128.9 ± 1.4
5.0	131.9 ± 1.8
100.0	17.1 ± 0.19
